# Intake of Common Alcoholic and Non-Alcoholic Beverages and Breast Cancer Risk among Japanese Women: Findings from the Japan Collaborative Cohort Study

**DOI:** 10.31557/APJCP.2020.21.6.1701

**Published:** 2020-06

**Authors:** Siamala Sinnadurai, Satoe Okabayashi, Takashi Kawamura, Mitsuru Mori, Nirmala Bhoo-Pathy, Nur Aishah Taib, Shigekazu Ukawa, Akiko Tamakoshi, The JACC Study Group

**Affiliations:** 1 *Kyoto University Health Service, Kyoto, Japan. *; 2 *Department of Social and Preventive Medicine, Faculty of Medicine, University of Malaya, Malaysia. *; 3 *Department of Population Medicine and Civilization Disease Prevention, Medical University of Bialystok, Poland. *; 4 *Hokkaido Chitose College of Rehabilitation, Chitose, Japan.*; 5 *Julius Centre University of Malaya, Faculty of Medicine, University of Malaya, Malaysia. *; 6 *Department of Surgery, Faculty of Medicine, University of Malaya, Malaysia. *; 7 *University Malaya Cancer Research Institute, Malaysia. *; 8 *Department of Public Health, Hokkaido University Graduate School of Medicine, Sapporo, Japan. *; 9 *Research Unit of Advanced Interdisciplinary Care Science, Graduate School of Human Life Science, Osaka City University, Osaka, Japan. *

**Keywords:** Beverages, breast cancer, risk, cohort study

## Abstract

This study investigated the association between intake of common alcoholic and non-alcoholic beverages and breast cancer risk among Japanese women. This study included 33,396 Japanese women aged 40–79 years from 24 areas in Japan from the Collaborative Cohort study. During the follow-up period (≥20 years), 245 incidents or mortal breast cancers were documented. Multivariable logistic regression analysis was performed to assess the independent association between breast cancer risk and the intake of Japanese green tea, coffee, and alcohol. Japanese green tea was the most commonly consumed non-alcoholic beverage (81.6% of participants), followed by coffee (34.7%) and alcohol (23.6%). No significant associations were identified between the intake of green tea and coffee with breast cancer risk (odds ratio OR 1.15, 95% confidence interval [CI] 0.82–1.60, and OR 0.84, 95% CI 0.64–1.10, respectively). Alcohol intake was associated with significant breast cancer risk (OR 1.46, 95% CI 1.11–1.92), and even infrequent alcohol consumption (<1 times/week) was associated with substantially increased breast cancer risk (OR 2.07, 95% CI 1.39–3.09). Alcohol type, especially, wine and whisky intake tended to be marginally associated with breast cancer risk (OR 1.79, 95% CI 0.99–3.23 and [OR] 1.68, 95% CI 0.91–3.08, respectively). Alcohol consumption would be associated with increased breast cancer risk. However, intake of green tea or coffee does not appear to be associated with increased breast cancer risk.

## Introduction

Breast cancer is the most common female malignancy and results in substantial morbidity and mortality worldwide (Bhoo Pathy et al., 2010). Compared with the United States and Europe countries, Japan have relatively low level of breast cancer mortality rates previously, however it has been increasing rapidly (Matsuda et al., 2014). This constant increase in breast cancer incidence was acknowledged as an important public health concern in Japan. While drinking habits were considered as prominent lifestyle risk factor of breast cancer, therefore it worth further investigation. 

Green tea is the most commonly consumed and been a routine diet beverage among Japanese. Epicatechin derivatives in green tea, has been hypothesized to have notable anti-carcinogenic effects. It was demonstrated in an animal experiments that drinking green tea can reduce the risk of developing breast cancer (Baligaet al., 2005). Contrasting viewpoints reported on association between green tea intake and breast cancer risk, lead to uncertain conclusions (Najafet al., 2018). Methodological issues, such as differences in study design and variations in exposure, may underlie this inconsistency.

While coffee is known as the most consumed beverage worldwide, the lowest intake has been reported among people in Eastern Asia (Oh et al., 2015). However, over the past 50 years, the Japanese population has been adapting to Western lifestyles in terms of dietary intake (Iwai et al., 2002), resulting in high rate of coffee consumption. Knowing that, coffee contains many components, including caffeine and polyphenols, it may play a dual role as a carcinogen (due to caffeine) by enhancing cell proliferation and as a chemopreventive agent (due to polyphenols) with antioxidative and weak estrogenic properties (Lee and Zhu, 2006; Olsen et al., 2004). A study conducted among premenopausal Japanese women showed that intake of caffeine-containing beverages was associated with the risk of developing breast cancer (Chisato et al., 1998). In contrast, a dose-response meta-analysis on prospective cohort studies reported coffee consumption was associated with decreased risk of postmenopausal breast cancer (Lafranconiet al., 2018). Nevertheless, epidemiological studies outcomes examined the associations between coffee consumption and breast cancer risk, the results remain inconclusive (Li et al., 2013).

Alcohol consumption is a well-established risk factor of breast cancer, also supported by a meta-analysis including 53 studies (Hamajima et al., 2002). Previous Japanese cohort studies reported no significant relationship between alcohol consumption and breast cancer risk (Goodman et al., 1997; Hirayama, 1991), but most recent cohort studies have revealed the higher alcohol consumption associated with increased breast cancer risk (Lin et al., 2005; Suzuki et al., 2010; Nitta et al., 2015). This inconsistency in the results may be related to the amount of alcohol consumption: Japanese women consume a low amount of alcohol compared to Western women, the difference stemming from a Japanese social stigma surrounding alcohol consumption (Nagata et al., 2007). Moreover, Japanese women consume particular kinds of alcoholic drinks, such as sake and shochu;the former is known as Japanese wine, made by fermenting rice and the latter is a spirit distilled from rice, barley, sweet potatoes, etc. However, so far there is no evidence on the association of type of alcoholic beverages consumed and breast cancer risk. 

Since green tea, coffee, and alcohol are important dietary exposures in the Japanese population, any association between the consumption of these beverages and breast cancer risk could have major public health impacts. Therefore, this study aimed to investigate the associations between the consumption of common alcoholic and non-alcoholic beverages and breast cancer risk among Japanese women using the data from a large prospective cohort study in Japan. 

## Materials and Methods

*Study population and assessment*

This study used the database of the Japan Collaborative Cohort Study for Evaluation of Cancer Risk (JACC study), which is a nationwide, multicenter, population-based, prospective cohort study to evaluate the risk of cancer incidence and mortality, sponsored by the Ministry of Education, Science, Sports and Culture of Japan. Details of the study have been described elsewhere (Ohno and Tamakoshi, 2001; Tamakoshi et al., 2013). Briefly, the JACC study was initiated in 1988, and the enrolment of subjects continued for two years until 1990. The original cohort included 110,585 (46,395 men and 64,190 women) participants aged 40–79 years who were recruited in 45 areas throughout Japan and underwent general health check-ups periodically, provided by the Japanese municipalities (Tamakoshi et al., 2013). Study participant’s health details were updated until 2009, unless they had moved out from their domicile or developed one of the endpoints determined in advance.

*Exposure assessment*

Information on beverage intake and other lifestyle factors was obtained using a self-administered questionnaire. For green tea and coffee, the participants were asked to report the frequency and amount of intake (daily, 3–4 cups per week, 1–2 cups per week, or 1–2 cups per month). For alcohol consumption, subjects were asked to report their drinking status (never, past, or current drinker). For current drinkers, the frequency of alcohol consumption (less than once a week, 1–2 times per week, 3–4 times per week, or daily), amount of alcohol per drink, and types of alcoholic beverages (sake, beer, whisky, wine, or others, multiple answers allowed) they preferred were further interrogated. 

*Follow-up and ascertainment of breast cancer occurrence*

The study outcome was incidence of breast cancer as well as deaths from breast cancer without previous breast cancer documentation. Data on cancer incidence, such as date of diagnosis and primary site, were collected only in 24 of the 45 study areas through prefectural population-based cancer registries or by reviewing the records of local major hospitals. The vital status of the participants was confirmed by reviewing death certificates with the permission of the Director General of the Prime Minister’s Office until the end of 2009, except for certain areas where follow-ups were discontinued. Both breast cancer death and incidence were treated as incidence because some participants had death records without incidence records.. 

*Statistical Analysis*

Potential risk factors were dichotomized to ensure sufficient statistical power. As for missing values, mean values were imputed for continuous variables, and the most likely categories were applied for categorical variables.

Logistic regression analyses were performed to estimate odd ratios (ORs) and their 95% confidence intervals (CIs) for the incidence of breast cancer according to the categories of the exposure variable and to adjust for confounding variables. Univariable analyses were performed to screen for potential independent predictors of breast cancer. In the subsequent multivariable analysis, established or biologically essential and statistically significant risk factors were adjusted in the model to investigate the independent association of each factor with the outcome. We considered the following variables to be potential confounders: age at baseline (40-59 vs 60-79 years old), education level (low<19 years old vs high≥19 years old), body mass index (<25 kg/m^2^ vs ≥25 kg/m^2^), green vegetable intake (< 3 times/week vs ≥ 3 times/week), red meat intake (< 3 times/week vs ≥ 3 times/week) , physical activity (≥ 1hour/week vs almost never), smoking habits (non-smoker vs smoker), parity (parous vs nulliparous), family history of breast cancer (no vs yes), and age at menarche (< 15 years old vs ≥15 years old). Age at menopause was not included in the model because the menopausal state was highly dependent on age. 

The order of the frequency category of alcohol intake was treated as a continuous variable for the test of linear trend. The absolute risk for each beverage was also calculated to find the true difference among beverages intake. The amount of daily ethanol intake was calculated by multiplying the total amount of alcohol use per occasion by the frequency of consumption divided by 7. The never drinking group of coffee, green tea or alcohol served as the reference, and an unknown status of drinking was excluded from the analysis. 

The analyses were performed using International Business Machines (IBM) SPSS statistics version 22.0. All tests of significance were two-tailed and p values of <0.05 or 95% CIs for ORs not including 1 were considered statistically significant. 

## Results

The present study included 40,466 women aged 40–79 years from 24 areas where cancer incidence or mortality were reported. After excluding 250 women with a past history of breast cancer, 1,879 dropouts, and 4,941 women who gave no information on drinking status, the remaining 33,396 women were included in the analysis. Table 1 summarizes baseline demographic, lifestyle, and dietary factors by the type of beverage intake. Mean age at enrolment was 57.7 (standard deviation [SD], 10.0) years. Japanese green tea was consumed daily by 81.6% of the participants and was the most commonly consumed beverage, followed by coffee, consumed daily by 34.7% of the participants. In contrast, alcoholic beverages were consumed by only 23.6% of the participants. Coffee drinkers were slightly younger and had higher education levels than non-coffee drinkers. Moreover, they were less likely to be nulliparous and more likely to consume fatty foods. Compared to non-coffee drinkers, coffee drinkers tended to be smokers. Furthermore, compared with non-alcohol drinkers, current alcohol drinkers tended to be highly educated, more frequently used sex hormones, were slightly younger, and highly preferred fatty foods and smoking. 

During the follow-up from 1988 through 2009, a total of 255 incident cases of breast cancer were documented. Several established risk factors, i.e., high education level, body mass index ≥25, nulliparity, and family history of breast cancer, were significantly associated with breast cancer (not shown in table). The associations between the type of beverage and breast cancer risk are shown in Table 2. No significant association was observed between either green tea and coffee consumption and breast cancer risk (multivariable OR 1.15, 95% CI 0.82–1.60 and multivariable OR 0.84, 95% CI 0.64–1.10, respectively). In contrast, alcohol intake was found to be associated with a significantly increased breast cancer risk (multivariable OR 1.46, 95% CI 1.11–1.92). 

Since there was increased breast cancer risk for alcohol consumption, we further focused on the frequency of alcohol intake (Table 3). Compared with non-drinkers, drinkers who consumed alcohol less than once a week displayed 2.07 times increased breast cancer risk (95% CI 1.39–3.09), whereas no dose-response relationships were found. Ethanol intake was 16.2 ± 14.5 g/drink for consumption of <1 times/week, 17.7 ± 33.6 g/drink for 1–2 times/week, 16.6 ± 13.2 g/drink for 3–4 times/week, and 20.7 ± 21.6 g/drink for daily consumption (p < 0.001), though data was missing for 1,077 (54.6%), 739 (33.9%), 278 (19.9%), and 203 (11.3%) participants, respectively.

For type of alcohol intake on breast cancer risk, we calculated the ORs for the restricted set of alcohol consumers who had reported the usual type of alcoholic beverage consumed (N = 7,916) (Table 4). No significantly increased risk was found for sake (multivariable OR 1.42, 95% CI 0.85–2.35) and beer (multivariable OR 0.91, 95% CI 0.59–1.42). In contrast, wine and whiskey consumption were associated with almost two-fold increased breast cancer risk, compared to their respective non-consumption, (multivariable OR 1.79, 95% CI 0.99–3.23 and multivariable OR 1.68, 95% CI 0.91–3.08, respectively) but not statistically significant. Among the alcohol consumers, the mean amount of logarithmically transformed ethanol intake was 0.74 ± 0.56 g/day for sake, 0.65 ± 0.54 g/day for beer, 0.62 ± 0.52 g/day for wine, and 0.87 ± 0.52 g/day for whisky.

The absolute risk of consumption for the various beverages was calculated as 0.01% (95% CI -0.13–0.33) for green tea, -0.09% (95% CI -0.28–0.11) for coffee, and 0.29% (95% CI 0.06–0.53) for alcoholic beverages for this cohort. Thus, alcohol intake yielded a small but significant absolute risk of breast cancer. For alcoholic beverages, the absolute risk was calculated as 0.42% (95% CI -0.00–0.88) for sake, 0.28% (95% CI -0.03–0.59) for beer, 1.11% (95% CI 0.17–2.05) for wine, and 1.05% (95% CI 0.11–1.99) for whiskey.

**Table 1:** Baseline Characteristics of the Study Participants by Beverage Type

	Overall	Green tea intake		p value^d^	Coffee intake		p value^d^	Alcohol intake			p value^e^
		Daily drinker	Non-drinker		Daily drinker	Non- drinker		Non-drinker	Past-drinker	Current-drinker	
Total participants, n (%)	33,396	27259 (81.6)	6137 (18.4)	-	11601 (34.7)	21795 (65.3)	-	24939 (74.7)	585 (1.8)	7872 (23.6)	-
Age, years, mean ± SD	57.7 ± 10.0	56.4 ± 10.1	58.0 ± 9.9	<0.001	55.0 ± 9.8	59.1 ± 9.8	<0.001	58.2 ± 10.0	59.0 ± 9.8	55.9 ± 9.8	<0.001
Education, high^a^, n (%)	3095 (10.8)	2582 (11.0)	513 (10.1)	0.08	1264 (12.2)	1831 (10.0)	<0.001	2221 (10.5)	51 (10.8)	823 (11.7)	0.03
Body mass index, kg/m^2^, mean ± SD	22.9 ± 3.3	22.9 ± 3.3	23.0 ± 3.2	0.04	22.8 ± 2.9	22.9 ± 3.4	0.03	22.9 ± 3.4	23.0 ± 3.2	22.9 ± 3.0	0.68
Nulliparous,n (%)	1464 (4.7)	1240 (4.9)	224 (3.9)	0.002	379 (3.5)	1085 (5.3)	<0.001	1073 (4.6)	47 (8.9)	344 (4.7)	<0.001
Use of sex hormone ^b^, n (%)	1480 (5.1)	1174 (5.0)	306 (5.7)	0.02	570 (5.5)	910 (4.9)	0.03	966 (4.5)	42 (7.9)	472 (6.8)	<0.001
Family history of breast cancer ^c^, n (%)	515 (1.5)	423 (1.6)	92 (1.5)	0.76	178 (1.5)	337 (1.5)	0.93	366 (1.5)	15 (2.6)	134 (1.7)	0.04
Age at menarche, years, mean ± SD	14.9 ± 1.8	14.9 ± 1.8	14.7 ± 1.8	<0.001	14.6 ± 1.8	15.0 ± 1.8	<0.001	14.9 ± 1.8	15.2 ± 2.0	14.7 ± 1.8	<0.001
Total dietary fiber intake, g/day, mean ± SD	12.0 ± 3.4	12.2 ± 3.4	11.2 ± 3.3	<0.001	11.5 ± 3.3	12.3 ± 3.4	<0.001	12.2 ± 3.3	11.0 ± 3.5	11.6 ± 3.5	<0.001
High preference of fatty food,n (%)	4473 (14.7)	3619 (14.5)	854 (15.2)	<0.001	1888 (17.3)	2585 (13.2)	<0.001	3015 (13.3)	84 (16.8)	1374 (18.6)	<0.001
Physical activity, ≥1 h/week, n (%)	7373 (23.9)	6129 (24.5)	1244 (21.3)	<0.001	2485 (22.8)	4888 (24.5)	<0.001	5172 (22.5)	143 (26.2)	2058 (28.2)	<0.001
Current smoker, n (%)	1568 (5.0)	1218 (4.8)	350 (6.1)	<0.001	857 (8.0)	711 (3.4)	<0.001	778 (3.3)	103 (20.3)	687 (9.6)	<0.001
Past smoker, n (%)	473 (1.5)	378 (1.5)	95 (1.7)	<0.001	206 (1.9)	267 (1.3)	<0.001	217 (0.9)	53 (10.4)	203 (2.8)	<0.001

SD, standard deviation; ^a^, Continuing education after high school graduation; b, Use of exogenous hormone or hormone replacement therapy; ^c^, Family history of breast cancer: history of breast cancer of participant’s mother, father, sister, or brother; ^d^ , Standard t-test or Mann Whitney-U test; ^e^, Analysis of variance or Kruskal-Wallis H test ;

**Table 2.** Association between Types of Beverage Consumed and Breast Cancer Risk

	Participants (n = 33,396) n (%)	Breast cancer (n = 255) n	Univariable OR (95% CI)	Multivariable OR^a^ (95% CI)
Green tea consumption				
Non-daily drinker	6,137 (18.4)	42	1	1
Daily drinker	27,259 (81.6)	213	1.14 (0.82–1.59)	1.15 (0.82–1.60)
Coffee consumption				
Non-daily drinker	21,795 (65.3)	173	1	1
Daily drinker	11,601 (34.7)	82	0.89 (0.68–1.16)	0.84 (0.64–1.10)
Alcohol consumption				
Non-drinker	24,939 (74.7)	172	1	1
Past-drinker	585 (1.7)	5	1.24 (0.51–3.03)	1.23 (0.50–3.03)
Current-drinker	7,872 (23.6)	78	1.44 (1.01–1.89)	1.46 (1.11–1.92)

OR, odds ratio; CI, confident interval; ^a^, Adjusted for age at baseline, educational level, body mass index, green vegetable intake, red meat intake, physical activity, smoking habits, parity, family history of breast cancer, age at menarche, green tea intake, coffee intake and alcohol intake

**Table 3:** Association between Frequency of Alcohol Consumption and Breast Cancer Risk

Frequency	Participants *n*	Breast cancer cases *n*	Univariable OR (95% CI)	Multivariable OR^a^ (95% CI)	*p* for trend
Non-drinker	24,939	172	1	1	
Past drinker	585	5	1.24 (0.51–3.03)	-	
<1 times/week	1,971	29	2.15 (1.45–3.20)	2.07 (1.39–3.09)	0.25
1–2 times/week	2,177	17	1.13 (0.69–1.87)	1.13 (0.68–1.87)	
3–4 times/week	1,399	12	1.25 (0.69–2.24)	1.24 (0.69–2.25)	
Daily	1,789	17	1.38 (0.84–2.28)	1.43 (0.86–2.37)	

OR, odds ratio; CI, confident interval; *p* for trends was calculated for alcohol intake patterns without past drinkers; ^a^, Adjusted for age at baseline, educational level, body mass index, green vegetable intake, red meat intake, physical activity, smoking habits, parity, family history of breast cancer, age at menarche, green tea intake, and coffee intake

**Table 4:** Association between Type of Alcohol Consumption and Breast Cancer Risk

Type of alcoholic beverage to drink frequently	Participants *n*	Breast cancer cases * n*	Univariable OR (95% CI)	Multivariable OR^a ^ (95% CI)
Sake				
No-drinker	24,939	172	1	1
Yes	2159	24	1.62 (1.05–2.49)	1.42 (0.85–2.35)
Beer				
No-drinker	24,939	172	1	1
Yes	4232	41	1.41 (1.00–1.98)	0.91 (0.59–1.42)
Wine				
No-drinker	24,939	172	1	1
Yes	777	14	2.64 (1.53–4.58)	1.79 (0.99–3.23)
Whiskey				
No-drinker	24,939	172	1	1
Yes	748	13	2.55 (1.44–4.50)	1.68 (0.91–3.08)

OR Odds ratio, CI confident interval; ^a^, Adjusted for age at baseline, educational level, body mass index, smoking habits, parity, and family history of breast cancer

## Discussion

This large prospective cohort study revealed intake of alcoholic beverages significantly increased breast cancer risk among Japanese women. The increased risk was observed for low-frequency intake (<1 time per week). Among alcoholic drinkers, wine and whiskey consumers presented with marginally increased breast cancer risk compared with non-drinkers. However, intake of Japanese green tea and coffee was not significantly associated with breast cancer risk. 

Regarding green tea, our findings supported the results of five other prospective cohort studies, including three Japanese studies (Dai et al., 2010; Inoue et al., 2008; Nagano et al.,2001; Suzuki et al., 2010; Suzuki et al., 2004), that reported no association between intake of green tea and breast cancer risk. Even though the large proportion of daily green tea drinkers (80%) weakened the statistical power, our study further strengthens past evidences (Nagano et al., 2001; Suzuki et al., 2010; Suzuki et al., 2004). 

Finding of coffee intake in our study seems to be compatible with those of other cohort studies conducted in Western settings (Fagherazzi et al., 2011; Ganmaa et al., 2008; Gierach et al., 2012; Ishitaniet al., 2008). A previous meta-analysis demonstrated no association between breast cancer risk and coffee consumption (Li et al., 2013), while two other meta-analyses reported statistically significant inverse associations between coffee consumption and breast cancer risk, depending on the women’s menopausal status (Jiang et al., 2013) and hormonal sensitivity of the tumors (Li et al., 2013). However, our study could not examine these factors due to insufficient of data. There are few studies carried out in the regions of the world where consumption of green tea dominates over coffee, such as Japan, lending great importance to our findings. 

Alcohol consumption significantly increased breast cancer risk in this study, but the absolute risk was small. Subsequent to the report of the World Cancer Report Fund in 2007, three cohort studies in Japan also demonstrated positive associations between alcohol consumption and risk of breast cancer (Lin et al., 2005; Nitta et al., 2015; Suzuki et al., 2010), in line with our findings. In addition, our results were also consistent with a large European cohort study and a meta-analysis conducted in Western populations (Hamajima et al., 2002; Tjonneland et al., 2007), although drinking patterns and types and amounts of alcohol intake largely differed from those of Japanese women. Our findings can be explained by the actions of estrogen and alcohol metabolism on breast cancer. Alcohol consumption substantially increases estrogen concentrations, and estrogens have a carcinogenic effect on breast cancer (Chen et al., 2011). Alcohol metabolites, such as acetaldehyde and lipid peroxides-reactive oxygen species, are also known as carcinogens (Wechsler et al., 1994), which may increase breast cancer risk. Furthermore, drinking patterns such as heavy episodic or binge drinking, leading to larger amounts of alcohol consumed in shorter periods of time (Morch et al., 2007), are known to elevate the levels of endogenous estrogen and lead to an accumulation of carcinogenic derivatives (Willett et al., 1987). There is a possibility that the majority of binge drinkers in our cohort study were classified into the group with intake <1 times/week. In this study, an attempt was made to calculate the amount of alcohol intake (g/ml) in each frequency category; however, a lot of data were missing for amount of alcohol consumed, especially in the low frequency group, so we could not accurately determine the amounts of alcohol consumed by the low-frequency drinking group.

As concerns the types of alcoholic beverages consumed, the habitual intake of sake and beer had no association with the risk for breast cancer, whereas that of wine and whiskey showed marginal increases in breast cancer risk, albeit not statistically significant. Additionally, we observed a small significant absolute risk in both wine and whiskey. The amount of ethanol consumed was similar between the different types of alcoholic beverages, except for whisky, which contributed more to ethanol intake than other types of alcoholic beverages. Several previous studies (Garland et al., 1999; Le et al., 1984; Longnecker, 1994) reported significant associations for beer, wine and liquor with breast cancer, while others did not (Chen et al., 2016; Garland et al., 1999; Kropp et al., 2001; Zhang et al., 1999). Since the amount of ethanol intake was not very different with respect to type of alcoholic beverage, the differences in drinking styles, such as time of drinking, consumption of aperitifs or night caps, and type of food consumption (side dishes) accompanying the specific alcoholic beverages, may affect subsequent health status. Since wine and whiskey were not common beverages when the study participants were recruited, it is not surprising that only few participants reported consumption of these beverages, preventing us from drawing firm conclusions.

The key strength of our study was the relatively large number of participants involved and the adjustments made for most specific potential confounders. Since, as only few studies have investigated the type of alcohol consumed in relation to breast cancer risk among Asians, particularly Japanese women, our study adds further evidence in this area.

This study has several limitations. First, the data on beverage intake were based on self-reporting; hence, measurement biases were inevitable. Second, we could not update the exposure data during the follow-up period; the types of beverages consumed and drinking patterns would have changed during the 20-year follow-up period, which could attenuate the true associations. Third, data on the amounts of alcohol consumed were unavailable, especially for the group of low-frequency alcohol consumption. We should generalize our findings with caution, and further research is warranted to overcome these limitations. 

In conclusion, in a population-based, prospective cohort study in Japan, alcohol intake increased breast cancer risk, even when consumed at a low frequency. However, both green tea and coffee intake did not appear to be associated with breast cancer risk. 
